# Venous Tone is a Critical Determinant of Venous Valve Closure in the Mouse

**DOI:** 10.1093/function/zqaf052

**Published:** 2025-11-03

**Authors:** Michael J Davis, Philip D King

**Affiliations:** Department of Medical Pharmacology & Physiology, University of Missouri, Columbia, MO 65212, USA; Department of Microbiology & Immunology, University of Michigan, Ann Arbor, MI 48109, USA

**Keywords:** back leak, valve incompetence, feed-forward

## Abstract

Venous diseases commonly involve venous wall and/or valve dysfunction. Chronic venous dilation, a characteristic of varicose veins, can progress to the point where venous valve (VV) leaflets are pulled sufficiently apart that they no longer prevent back flow. Incompetent VVs increase the load on more distal valves by increasing the standing column of proximal blood. We tested VV function by isolating single valves from cervical veins of the mouse and measuring back leak and the adverse pressure gradient required for closure. Valve identification was facilitated by genetically forced expression of GFP in VV endothelium. A causal relationship was found between the relative diameter of the vein and VV closure, with a striking effect of venous tone: ∼60% of mature VVs in the cervical vein were incapable of closing if the vessel lost spontaneous tone and, in another ∼20% of veins, VVs closed only when venous tone exceeded some threshold value. Our results have important implications for the causes and possible treatment of VV incompetence in pathological states such as venous varicosity and chronic venous insufficiency. Moreover, they suggest an underappreciated mechanism whereby loss of venous tone can initiate a feed-forward cycle of events that make valve closure increasingly difficult, thereby elevating local venous pressure and exacerbating the loss of tone. This detrimental cycle may potentially be interrupted by appropriate pharmacological therapy to enhance venous tone and thereby restore VV competence.

## Graphical Abstract

**Graphical Abstract:**
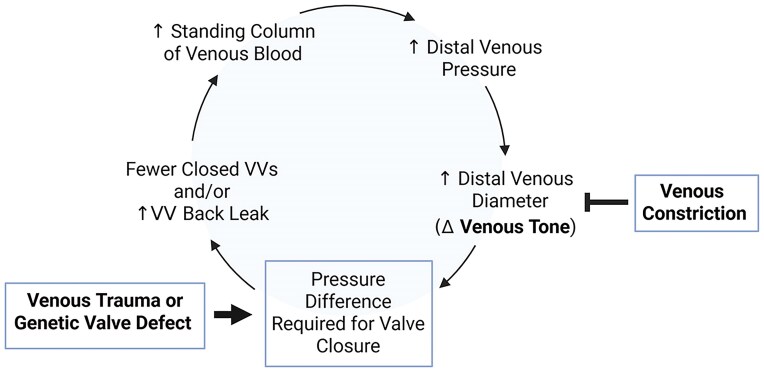
Mechanism by which loss of venous tone would lead to impaired venous valve (VV) closure and/or increased VV back leak in a particular vein, thereby increasing the standing column of venous blood and further impairing venous tone, which further compromises VV function. Created and licensed in BioRender. Davis, M. (2025) https://BioRender.com/u764qck

## Introduction

Venous valves (VVs) are important components of the venous system. The valves in major veins dictate unidirectional movement of venous blood, which is particularly important (1) during skeletal muscle contraction, when the blood in peripheral veins is squeezed and potentially forced to flow in either direction, and (2) during standing, when periodically spaced valves are needed to break up an otherwise continuous hydrostatic column of venous blood that would pool in the extremities.^1^ In rodents, both lymphatic and VVs are thin, bileaflet structures composed of 2 layers of endothelial cells surrounding an extracellular matrix core. The 2 leaflets are anchored at a common base in the vessel wall, with their outer edges extending downstream to insert into the wall and their free, inner edges forming an elliptical opening in the vessel lumen.^2,3^ The developmental stages and factors controlling VV development, including the expression of PROX1, are similar to those of lymphatic valves.^4^

Venous diseases are common^5^ and are almost always associated with venous wall and/or valve dysfunction.^6^ While normal VVs are mechanically robust and capable of withstanding substantial tensile stress,^7,8^ VV leaflets can become deformed or perforated, and their movements can be impaired by attached thrombi,^9^ thereby inducing various degrees of valve incompetence. Chronic venous dilation, characteristic of varicose veins, can progress to the point where VV leaflets are pulled sufficiently apart to no longer overlap and prevent back flow.^10^ Incompetent VVs will increase the load on more distal valves by increasing the standing column of proximal blood.

In the course of studying normal and pathologic lymphatic valves in rats and mice, we noted a distinct relationship between the relative vessel diameter and the adverse pressure gradient required for lymphatic valve closure.^11^ At low diameters (<40% of maximal passive diameter) an adverse pressure gradient (ΔP) <0.2 cmH_2_O is usually sufficient to induce valve closure; however, as the vessel distends to its maximal diameter, the ΔP for closure increases by 10 to 20-fold. This effect becomes more pronounced in pathological states in which valve leaflets partially regress.^12-16^

Here, we describe a similar relationship between the relative vein diameter and VV closure in mice but with an additional and striking effect of venous tone: ∼60% of mature VVs in the cervical vein are incapable of closing if the vessel loses spontaneous tone and, in another ∼20% of cervical veins, valves close only when tone exceeds some threshold value. Our results have important implications for the causes and possible treatment of VV incompetence in pathological states such as varicose veins and chronic venous insufficiency. Moreover, they suggest an underappreciated mechanism whereby loss of venous tone initiates a feed-forward cycle of events that make valve closure increasingly difficult, thereby elevating local venous pressure and exacerbating the loss of tone. This detrimental cycle may potentially be interrupted by appropriate pharmacological therapy to enhance venous tone and thereby restore VV competence.

## Methods

### Animal Protocols and Ethical Approval

All animal procedures were approved by the Animal Care Committee at the University of Missouri (protocol #9797) and complied with the standards stated in the “Guide for the Care and Use of Laboratory Animals” (National Institutes of Health, revised 2011). Mouse strains used for experiments included C57Bl/6 mice, obtained from JAX, *Prox1-GFP* mice, obtained from Young-Hong (USC) and *Rasa1^f/f^; Prox1-GFP* (without cre) mice. Mice of either sex, ages 3-8 months, were used for all protocols. For logistical reasons, the data represent pooled samples from male and female mice without regard to age.

### Vein Isolation and Cannulation

A mouse was anesthetized with ketamine/xylazine (100/10 mg/kg, i.p.) and placed on a heating pad. The skin on the upper chest was shaved and a ventral incision from the chin to the sternum made to expose the superficial cervical lymphatic vessels and underlying cervical and external jugular veins (EJV). After hydration with Krebs solution, the loose fascia swelled and was easily removed by fine scissors. The cervical vein, along with the EJV branch, was cut centrally and grasped with fine forceps while pulling it caudally and trimming the edges, until a segment 2-4 mm in length was obtained. In most cases, a VV was located in the cervical vein ∼0.5 cm distal to the EJV junction. Some segments also had 1-2 additional smaller tributary veins peripheral to the junction. After cutting the distal ends of these vessels and any branches, the entire structure was removed and placed in a dissection dish on a layer of SYLGARD (Dow Corning, Midland IN) bathed in room temperature Krebs-BSA solution containing: 146.9 m m NaCl; 4.7 m m KCl; 2 m m CaCl_2_·2H_2_O; 1.2 m m MgSO_4_; 1.2 m m NaH_2_PO_4_·H_2_O; 3 m m NaHCO_3_; 1.5 m m sodium-HEPES; 5 m m D-glucose; 0.5% BSA (pH 7.4 at 37°C).

After pinning down the segment with pieces of 40 μm wire, the remaining connective tissue and fat were removed and the segment was transferred to a 3 mL cannulation chamber. The proximal and distal ends were cannulated onto 60-100 μm glass micropipettes filled with Krebs-BSA and tied with 12-0 suture (GEM194BK, Synovis Micro Co., Birmingham, AL, USA). After pressurization, red blood cells were flushed out and any side branches were identified and ligated with 12-0 suture. The cannulation chamber, with attached pipette holders and vessel, was transferred to the stage of an inverted microscope, heated to 37°C and perfused with Krebs buffer (0.5 mL/min) using a peristaltic pump (Minipuls, Gilson). Polyethylene tubing connected the back of each micropipette to low pressure transducers (CyQ, Nicholasville, KY, USA) and a computerized 2-channel pressure controller (Cardiovascular Research Institute, Texas A&M University), driven by a LabVIEW program through a D-A interface (National Instruments, Austin, TX, USA) under Windows OS, allowing independent control of inflow (P_in_) and outflow (P_out_) pressures.^17^ Both pressures were briefly set to 10 cmH_2_O at the beginning of every experiment and the vessel segment was stretched axially to remove longitudinal slack. The segment was then equilibrated in Krebs buffer (without BSA) for 30 min at 2 cmH_2_O luminal pressure. Development of spontaneous tone confirmed vessel viability. The vessel image was digitized using a firewire camera (Basler, model A641FM) and inner diameter was continuously tracked using a custom computer algorithm.^18^ All protocols were recorded as AVI files, with pressure and diameter data embedded in the file, for later replay for diameter and/or valve tracking, as needed. VV tests were performed either in Krebs solution or Ca^2+^-free Krebs solution (Krebs solution with 3 m m EDTA replacing CaCl_2_·2H_2_O) to eliminate spontaneous tone. Tone was calculated as the % difference in vessel diameter in Krebs vs Ca^2+^-free Krebs solution at any given pressure.

### Valve Function Tests

After a vein segment was mounted on the microscope, and before tone developed, a 10-μm initial hole in the top surface of the vessel near the P_in_ pipette was made with a sharply tapered pilot micropipette, which was then removed and replaced with a more gradually tapered servo-null micropipette (tip diameter ∼5 μm) to measure luminal pressure on the inflow side of the valve (P_sn_). After insertion, the servo-null micropipette was advanced to seal the hole. The pipettes were fashioned from borosilicate glass (1.0/0.5 mm, ID/OD; Fredick Haer, Bowdoin, ME) on a Sutter *P*-97 puller (Sutter Instruments, Novato, CA, USA). The servonull system was an IPM model 4A (Instrumentation for Physiology & Medicine, La Jolla, CA, USA). The calibration of the servo-null pipette was checked and adjusted as needed after raising P_in_ and P_out_ simultaneously between 0.5 and 10 cmH_2_O. To ensure accurate and consistent measurements of valve back leak: (1) all three transducers (P_in_, P_sn_, P_out_) were calibrated before each experiment; (2) the P_sn_ pipette calibration was checked at the beginning and end of each valve test; and (3) the pipettes and cannulation tubing were free of bubbles and the pipette tip was free of debris. The pipettes were cleaned in distilled H_2_O and acetone after each experiment.

### Back Leak Measurement

After a vein developed spontaneous tone in Krebs solution at pressure = 2 cmH_2_O, pressure was lowered to 0.5 cmH_2_O. When tone had stabilized and with the valve open, P_out_ was raised, ramp-wise, to 10 cmH_2_O over a 35-sec period while P_in_ was held at 0.5 cmH_2_O. Valves typically closed as P_out_ exceeded ∼0.3 cmH_2_O and remained closed for the duration of the P_out_ ramp. In other cases, gentle tapping on the P_out_ line facilitated valve closure at the beginning of the pressure ramp. Pressure back leak through the closed valve was measured with the servo-null micropipette on the inflow side of the vessel, which could resolve changes as small as ∼0.05 cmH_2_O. Back leak was defined as P_sn_ at the end of the ramp minus the value of P_in_. The P_out_ ramp was repeated 3 times. Values of P_sn_ at intermediate P_out_ levels were determined offline using a LabView program that binned the P_sn_ data in 0.5 cmH_2_O intervals of P_out_ for plotting and statistical tests.

### Closure Test

A second test determined the adverse pressure gradient (ΔP, P_out_—P_in_) required to close an open valve. As demonstrated previously for lymphatic valves,^11,13^ this value increased with increasing vessel diameter. The measurements therefore were made over a wide range of baseline pressures, each of which impacted the baseline diameter. Starting with the valve open, P_out_ was raised, ramp-wise, and the ΔP was determined at the instant of valve closure. The test was repeated for baseline pressures 0.1, 0.2, 0.3, 0.5, 1, 2, 3, 5, 8, 10 cmH_2_O, resulting in tests over a range of diameters spanning ∼10% to 100% of the maximal diameter (Dmax) when P_in_ = 10 cmH_2_O, determined either in Krebs or Ca^2+^-free Krebs solution. ΔP for closure was then plotted against the normalized diameter (D/Dmax). The highest ΔP that could be tested was 30 cmH_2_O (equating to a maximum P_out_ of 40 cmH_2_O when P_in_ was 10 cmH_2_O) without exceeding the specified safe range of the pressure sensor elements.

Valve function tests were first conducted in Krebs solution, in which each cervical vein developed a variable amount of spontaneous tone. The tests were then repeated on the same vessel/valve after elimination of tone in Ca^2+^-free Krebs solution (for >20 min). Paired tests were made on 14 vessels from 10 mice. In 11 additional vessels from 7 mice, no valid measurements or only partial measurements could be made due to various technical problems (pipette plugging, leak developing, etc.) and those data were not used for analysis of valve function.

## Results

### Regional Variations in Murine VVs

**Figure 1.**
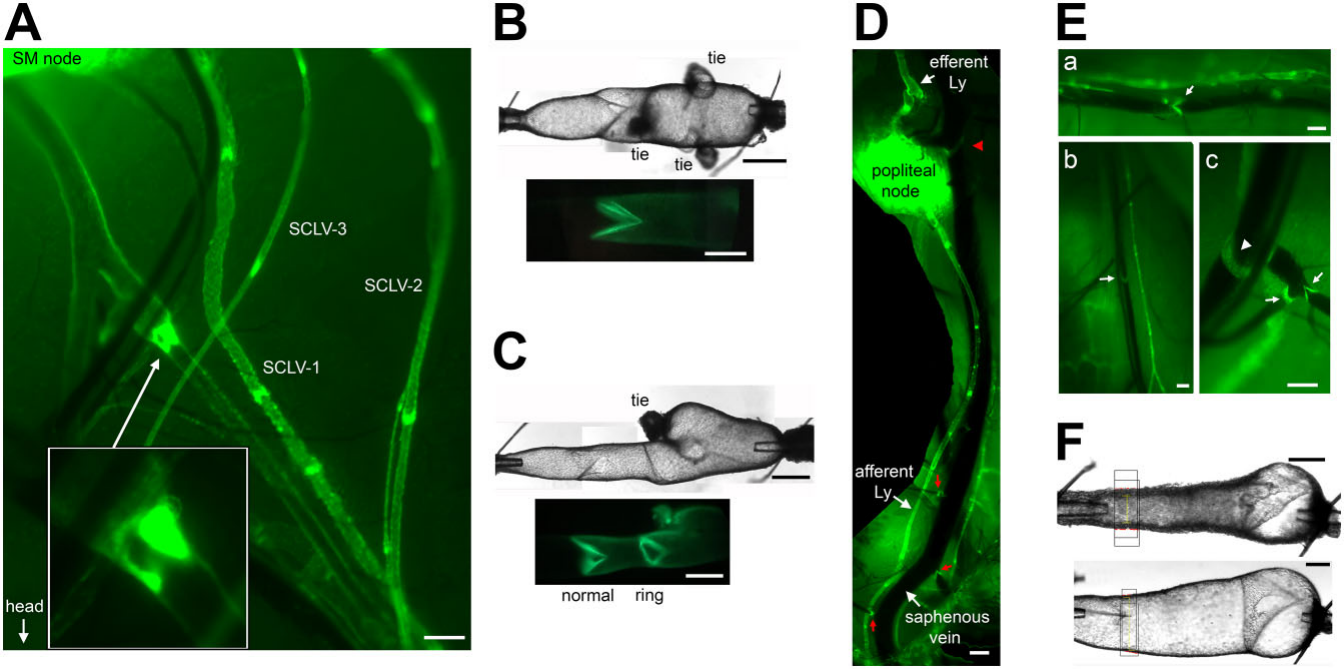
Images of VVs in various veins of *Prox1GFP* mice in situ and *ex vivo*. (A) *In situ* fluorescence image of the junction of cervical and external jugular veins in the neck of a *Prox1-GFP* mouse, showing the location of a GFP + valve in the cervical vein. The smaller GFP + vessels with numerous valves are the three superior cervical lymphatics (SCLV-1, SCLV-2, SCLV-3) that drain into the submandibular (SM) node.^34^ The lymphatic vessels normally overlie the vein but in this image they have been pulled to the side to expose the venous valve (insert). (B) Brightfield (top) and fluorescence (bottom) images of an isolated cervical venous valve preparation showing the inflow pipette (left) and outflow pipette (right). Three side branches were tied in order to maintain pressure control. In the corresponding fluorescence image, the GFP + venous valve and downstream field of GFP + LECs are visible. (C) Brightfield (top) and corresponding fluorescence (bottom) images of another cervical vein containing 2 valves. The valve at the left has 2 fully formed leaflets while the valve at the right is a non-functional “ring” valve. (D) Infrequently, a VV was present in the saphenous vein near the popliteal node (red arrowhead). VVs were more common in small tributaries of the saphenous vein (red arrows), but upon close inspection, many of those valves appeared to be in the “ring” stage,”^20^ ie, nonfunctional. (E) Fluorescence images of (a) a valve in the lower saphenous vein at the level of the ankle, (b) a single venous valve in a brachial vein alongside a lymphatic collector, (c) a valve in another brachial vein (arrowhead) and 2 additional valves in its venous tributaries (arrows). (F) Images of an isolated (unbranched) cervical vein with tone (top) in Krebs solution and without tone (bottom) in Ca^2+^-free Krebs solution. All scale bars = 200 μm.

The major VVs in the mouse, located in only a few regions, have been described previously.^19^ In the present study, expression of the GFP reporter in PROX1 + cells facilitated identification of VVs, variations in their locations, and the presence of additional valves in tributaries of the major veins. VVs in the femoral and cervical veins were present in all animals studied. Femoral vein valves were located at the confluence of multiple tributaries (sometimes 5-6 smaller vessels) and therefore were not amenable to isolation, cannulation and controlled pressurization. In one mouse, the proximal saphenous vein contained a VV just prior to joining the femoral vein, but *ex vivo* tests of that valve revealed it to be incompetent (ie, it would not close under an imposed adverse pressure gradient of 30 cmH_2_O). Therefore, we focused our attention on the cervical vein, where a single VV was typically present just upstream from its junction with the EJV (Figure 1A-B). In 5 out of 25 vessels, a second valve was also located in the cervical vein within 0.5 cm upstream or downstream from the fully formed valve (Figure 1C), but in all 5 cases the second valve was an incompetent, non-functional ring valve, as described previously in developing lymphatic vessels.^20^ In a few mice, a VV was present in the saphenous vein near the popliteal node (red arrowhead, Figure 1D), but this valve occurred too seldom to be systematically studied; VVs were more common in small tributaries of the saphenous vein, immediately at their junctions with the larger vein, but these smaller veins (red arrows, Figure 1D) could not routinely be cannulated. A single VV was often present in the lower saphenous vein near the ankle (Figure 1Ea), but usually in proximity to multiple small side branches that were difficult to ligate, making valve tests impractical. In most but not all mice, a single VV was also present in the brachial vein (Figure 1Eb), and/or in its tributaries (Figure 1Ec). However, in preliminary studies of brachial VVs, none of those valves closed under an adverse pressure gradient of 30 cmH_2_O.

During dissection, cervical veins (and most of the other veins described above) reacted *in situ* to gentle touch with a fine forceps (direct or nearby) with strong, transient constrictions, often to closure or near-closure. After isolation, cannulation, pressurization, and equilibration in Krebs solution at 36-37°C, cervical veins developed variable degrees of spontaneous tone (Figure 1F). Tone was particularly pronounced at pressures <2 cmH_2_O, which is likely within the normal operating range of these vessels *in vivo*.

**Figure 2.**
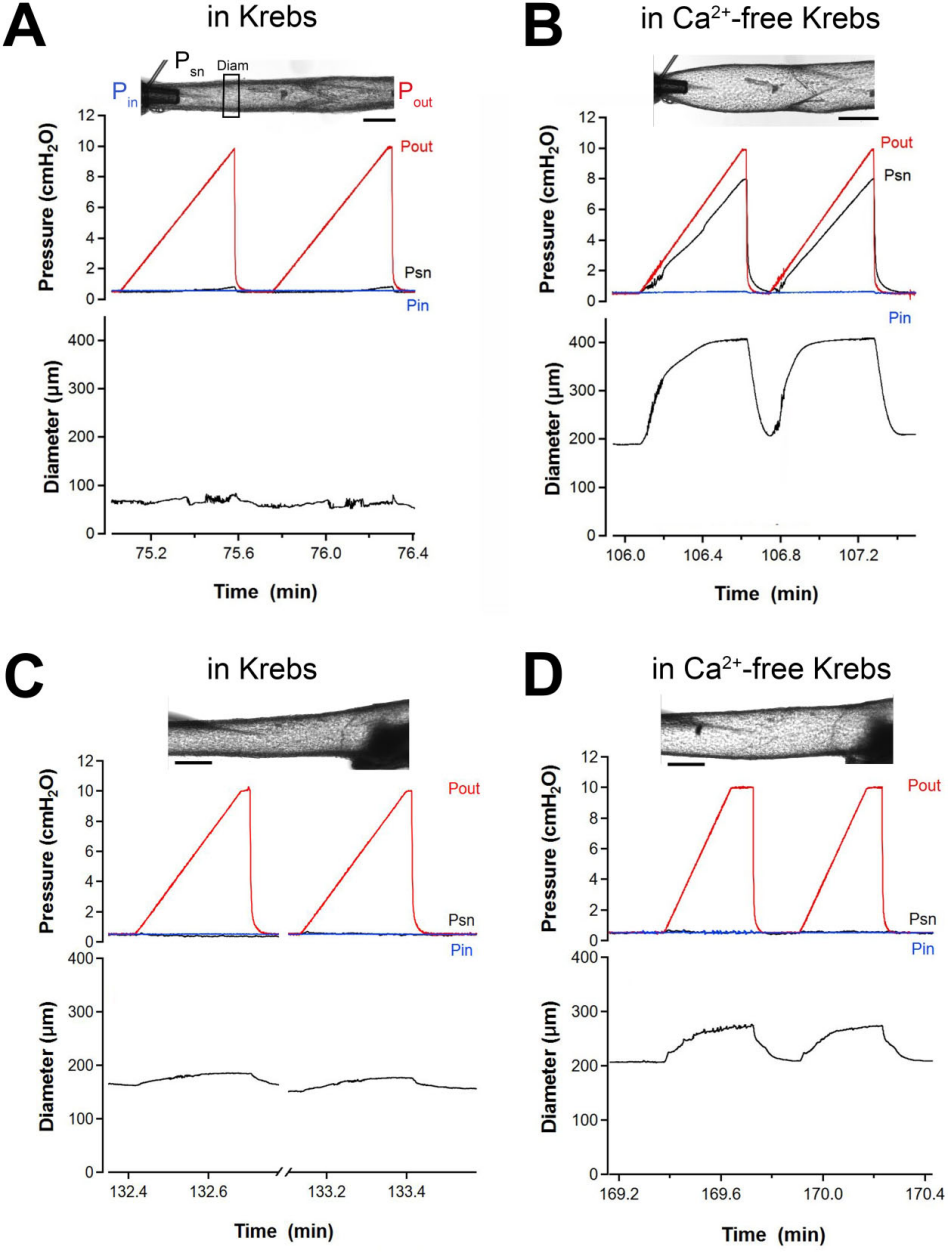
Back leak tests for cervical VVs with and without tone. Examples of back leak tests across isolated VVs in 2 cervical veins. In Krebs solution, the first vessel developed 62% spontaneous tone and the valve closed, preventing an increase in P_sn_ when P_out_ was raised (A), but when tone was eliminated after 20 min equilibration in Ca^2+^-free Krebs solution the valve no longer closed (P_sn_ rose from 0.5 to 8 cmH_2_O) upon P_out_ elevation (B). Insets show images of the vessel with and without tone. In the left image, the inflow cannulating pipette (P_in_), outflow pipette (P_out_) and servo-null pipette (P_sn_, with tip in the vessel lumen) are shown. The tracking window shows where internal diameter (Diam) was measured upstream of the valve. Each P_out_ ramp was repeated twice. The second vessel (lower panels) developed 22% tone in Krebs solution and the valve closed (ie, P_sn_ remained at 0.5 cmH_2_O) when P_out_ was raised (C). In contrast to the first vessel, the valve continued to close (ie, P_sn_ did not rise) during the P_out_ ramp even after tone was eliminated in Ca^2+^-free Krebs solution (D). Each P_out_ ramp was repeated twice. All scale bars = 200 μm.

We conducted 2 related but different tests of VV function. Back leak tests used a servo-null micropipette to measure the pressure back leak across a closed valve. Each valve was initially open at the beginning of the test but closed when P_out_ was raised ramp-wise. In previous studies of lymphatic valves, we conducted valve function tests starting at a pressure of 0.5 cmH_2_O. Following that precedent, we performed valve back leak tests on cervical VVs, with examples of 2 different behaviors shown in Figure 2. In Krebs solution, the diameter of the first vessel at equal pressures of 0.5 cmH_2_O was ∼71 μm upstream from the valve (62% tone). As P_out_ was raised ramp-wise to 10 cmH_2_O, with P_in_ held constant, only a small increase in diameter (to 79 μm) on the upstream side of the vessel occurred, an increase that was limited by a partially or completely closed valve (Figure 2A). P_sn_, also measured upstream from the valve, rose only from 0.5 to 0.8 cmH_2_O during the P_out_ ramp. This response was highly repeatable, as shown by the second back leak test in Figure 2A. The behavior of the same vessel/valve was strikingly different in the absence of tone. In Ca^2+^-free Krebs, the initial diameter was 188 μm at P_in_ = P_out_ = 0.5 cmH_2_O and rose to 406 μm when P_out_ was raised to10 cmH_2_O (Figure 2B). The valve never closed during the P_out_ ramp in Ca^2+^-free Krebs solution and P_sn_ rose from 0.5 to 8.0 cmH_2_O, indicative of an incompetent valve (the maximum P_sn_ value was determined by the resistances of the 2 canulating pipettes, the resistance of the vessel segment and the relative position of the servo-null pipette, as discussed previously).^21^ Although 8 out of 14 cervical vessel/valves behaved in the manner shown in Figure 2A-B, not all did. Another type of behavior, observed in 5 out of 14 vessels, is shown in Figure 2C-D. In those cases, the cervical VVs closed during the P_out_ ramps, preventing back leak, regardless of tone (ie, in both Krebs solution and Ca^2+^-free Krebs solution). In the example shown here, a second vein developed only 20% tone prior to beginning the test but P_sn_ did not change as P_out_ rose from 0.5 to 10 cmH_2_O in Krebs (Figure 2C) nor did P_sn_ change during an identical P_out_ ramp in Ca^2+^-free Krebs solution (Figure 2D). This behavior is similar to that of many lymphatic valves, for which back leak tests are typically conducted in Ca^2+^-free Krebs solution to eliminate interference from pressure spikes associated with spontaneous contractions. A third type of behavior was also noted in 1 out of 14 vessels, in which the valve would not close regardless of whether tone was present (not shown) but the data were nevertheless included in the summary analysis. Of the 14 cervical veins studied, the average tone in Krebs at equal pressures of 0.5 cmH_2_O was 63 ± 1% (S.E.M.).

**Figure 3.**
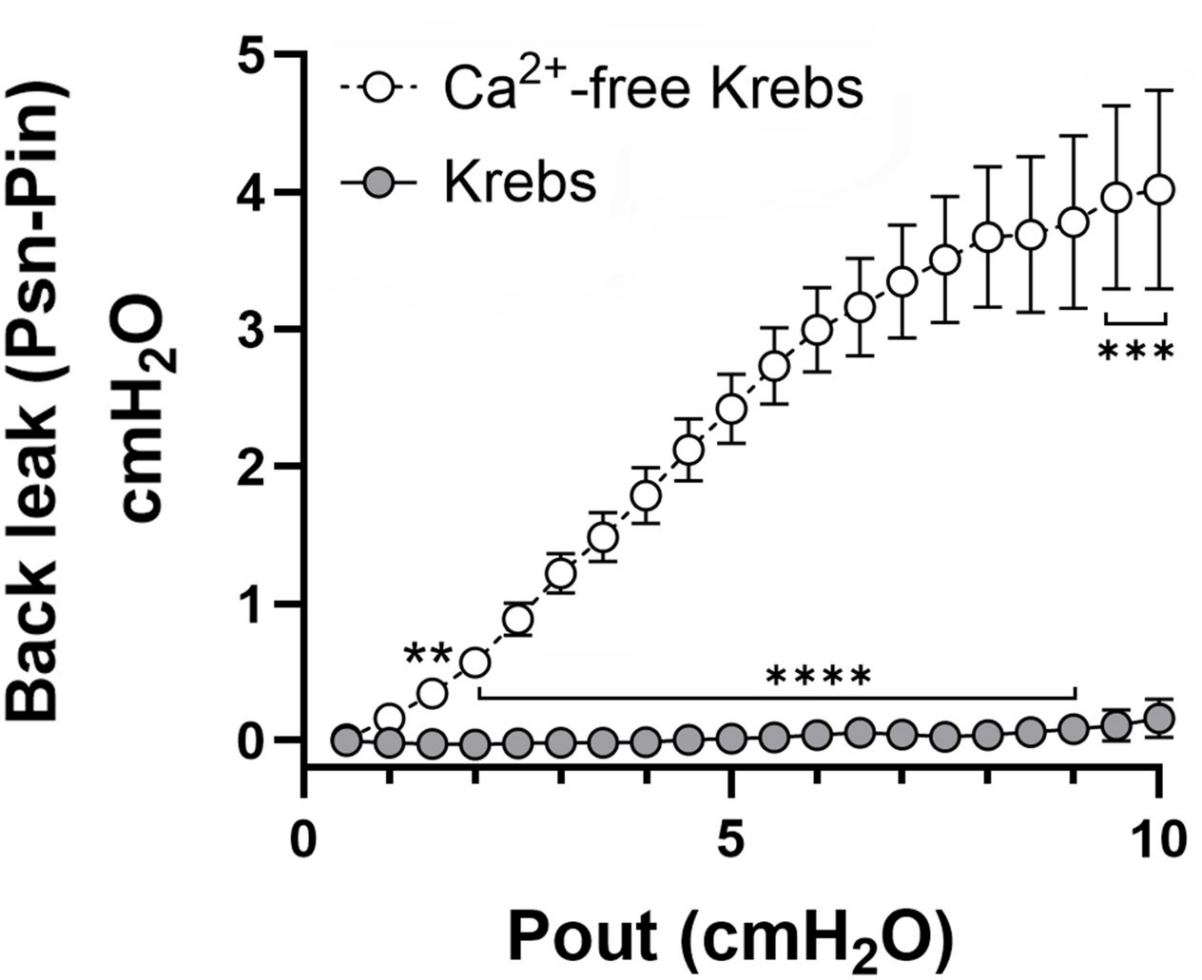
Summary data for back leak tests. Average values of P_sn_ measured upstream of isolated VVs during P_out_ ramps from 0.5 to 10 cmH_2_O. VVs closed and P_sn_ rose only slightly when vessels developed tone in Krebs solution (filled gray symbols), whereas P_sn_ rose to an average of ∼4 cmH_2_O during P_out_ ramps when tone was eliminated in Ca^2+^-free Krebs (filled white symbols), indicating that VVs did not close. Significance is designated by: ** *P* < 0.01, *** *P* < 0.001, **** *P* < 0.0001, as determined using a 2-way repeated measures ANOVA with Dunn’s multiple comparison post-hoc tests. Paired sets of vessels were used; *N* = 10, *n* = 14.

Average values of P_sn_ (minus the value of P_in_) measured upstream of VVs during P_out_ ramps are summarized in Figure 3. VVs closed and P_sn_ rose only slightly (from 0 to 0.2 cmH_2_O) when vessels developed tone in Krebs solution (filled gray symbols). This analysis included 10 valves with little or no back leak only when tone was present, 3 valves with little or no back leak regardless of tone and 1 valve with back leak regardless of tone. In contrast, when tone was eliminated in Ca^2+^-free Krebs, P_sn_ rose from 0 to an average of ∼4 cmH_2_O during P_out_ ramps to 10 cmH_2_O (filled white symbols), indicative of vessels with valves that did not close. The differences between the 2 groups were highly significant except at the lowest 2 levels of P_out_.

**Figure 4.**
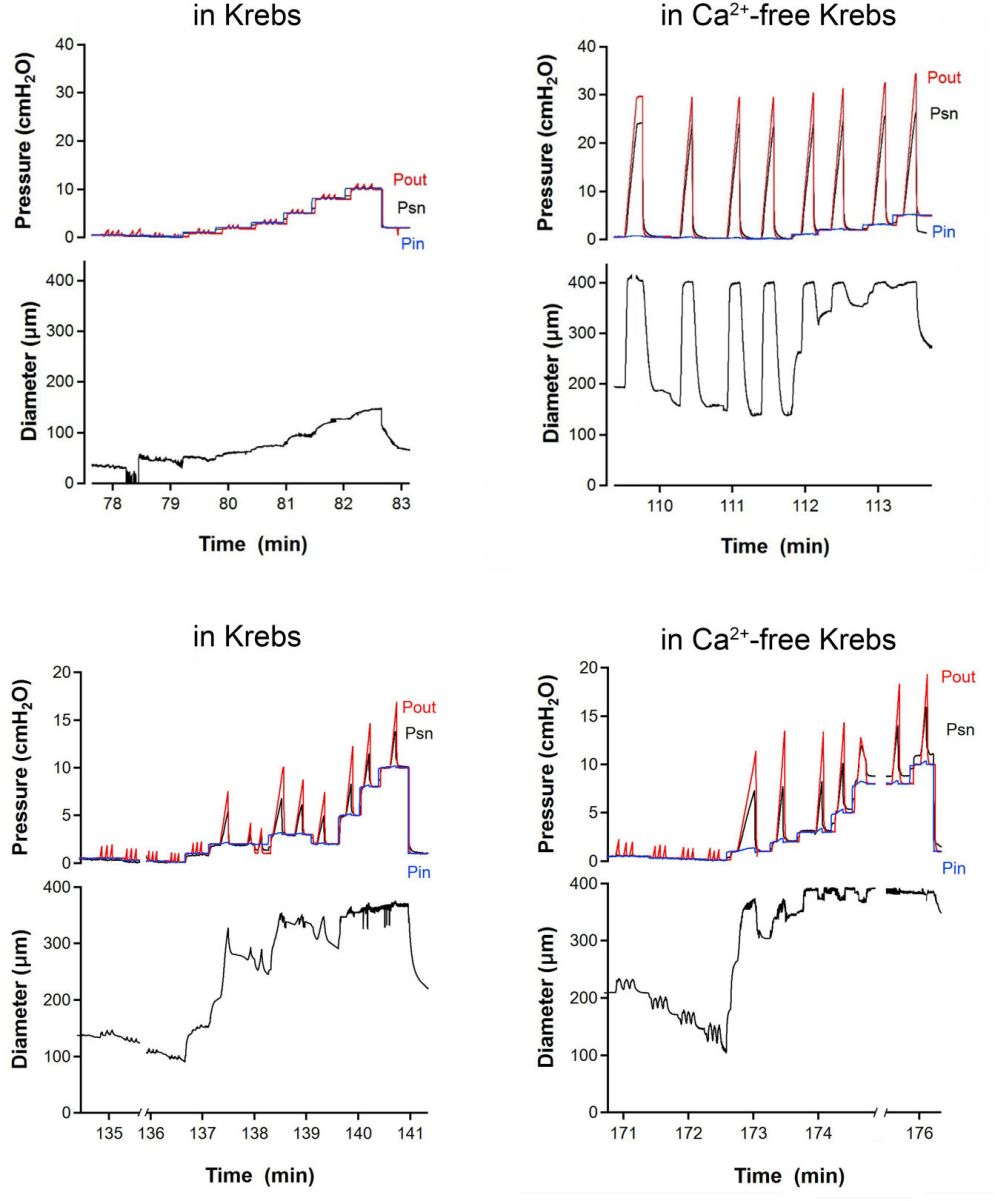
Closure tests for cervical VVs with and without tone. Examples of closure tests for isolated VVs in 2 cervical veins (same vessels/valves shown in Figure 2). (A) In Krebs solution, the first vessel developed 79% spontaneous tone when P_in_ and P_out_ were initially set to 0.5 cmH_2_O and the valve was open; P_out_ was then selectively raised until the valve closed [closure is evident both in the video image (not shown) and by a limited increase in P_sn_ (< 1 cmH_2_O)]. The adverse pressure gradient (ΔP) required for closure was calculated from (P_out_-P_in_) at the instant of valve closure. The test was repeated 1-3 times at various P_in_ levels from 0.1 to 10 cmH_2_O. (B) Closure tests on the same valve in Ca^2+^-free solution. In these cases, P_sn_ rose to > 25 cmH_2_O during each respective P_out_ ramp because the valve did not close. (C-D) Closure tests on a second VV that closed during each test regardless of whether tone was present. Note the different *y*-axis scales used in panels A-B and panels C-D. *N* = 10, *n* = 14.

We also conducted closure tests on each valve in the presence or absence of spontaneous tone (Figure 4). This test began with an open valve and measured the adverse trans-valve pressure difference required to close the valve, ie, the value of (P_out_-P_in_) at the instant of closure, as P_out_ was increasing. A maximal P_out_ value of 40 cmH_2_O (and ΔP of 30 cmH_2_O) was dictated by the overpressure limit of the low-pressure transducers in our servocontrol system. The P_sn_ measurement was not used in this test but a sharp drop in P_sn_, after it started to rise with P_out_, occurred when the valve closed and was a useful indicator of closure [see traces in Figure 3 of^21^] in cases where the valve was not optimally oriented for viewing under the microscope (as in Figure 2C). Examples of closure tests for isolated VVs in 2 cervical veins are shown in Figure 4A-B and Figure 4C-D, respectively. In Krebs solution, the first vessel developed 79% spontaneous tone when P_in_ and P_out_ were initially set to 0.5 cmH_2_O and the valve was open; P_out_ was then selectively raised until the valve closed [valve closure was evident both in the video image (not shown) and by an initial increase and then drop in P_sn_ when the valve closed]. The test was repeated 1-3 times at P_in_ levels = 0.3, 0.2, 0.1, 1, 2, 3, 5, 8, and 10 cmH_2_O, with ΔP for closure increasing at the higher levels of P_in_. Closure tests on the same valve in Ca^2+^-free solution are shown in Figure 4B. In that case, P_sn_ rose to > 25 cmH_2_O during each respective P_out_ ramp because the valve did not close. In contrast, the valve in a second vessel closed during each test regardless of whether the test was performed in Krebs (Figure 4C) or Ca^2+^-free Krebs (Figure 4D).

**Figure 5.**
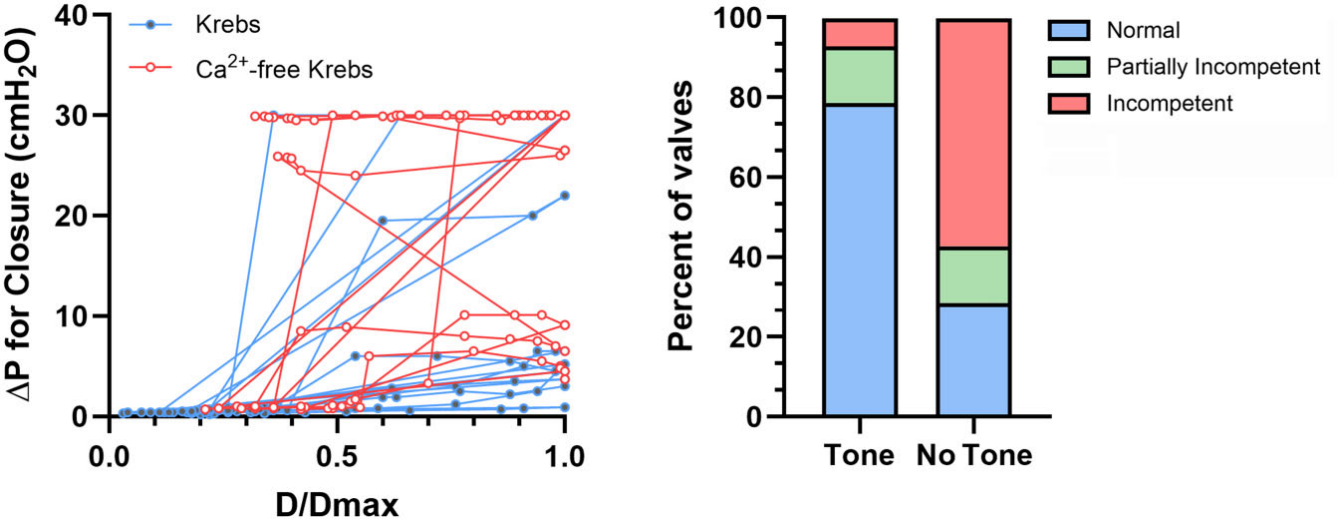
Valve closure tests. (A) The adverse ΔP required for closure plotted at the corresponding initial diameter determined at each level of P_in_ in Krebs solution (blue lines and filled gray symbols) or Ca^2+^-free Krebs solution (red lines and filled white symbols). Each connected line represents the data set for a single valve. If a valve did not close, Δ*P* = 30 cmH_2_O. (B) Percentage of normal (adverse Δ*P* < 10 cmH_2_O), partially incompetent and completely incompetent valves in cervical veins, with or without tone, with their classification based on the behavior shown in panel A.

Closure test data for 14 VVs are summarized in Figure 5. With tone, 11 of 14 valves closed under reasonable values of adverse ΔP (<7 cmH_2_O), even when the veins were “maximally” distended (D/Dmax = 1) at P_in_ = P_out_ = 10 cmH_2_O (Figure 5 panel A). With tone, 2 valves closed at lower diameters but became incompetent when their diameters exceeded values of 30 and 40% of maximum, respectively. A third valve was partially incompetent when its relative diameter exceeded 40% of maximum. In contrast, in the absence of tone, only 4 of 14 valves closed at reasonable values of adverse ΔP (<10 cmH_2_O), 7 were incompetent at all pressure/diameters, one was partially incompetent at all pressures/diameters and 2 became incompetent when diameters exceeded 40 and 70% of the maximum passive diameter. Panel B of Figure 5 summarizes the percentage of valves that were normal, partially or completely incompetent in the presence or absence of tone.

## Discussion

### Venous Tone Controls VV Function

Our study is the first to demonstrate a relationship between venous tone and whether a VV will be competent. In previous studies of rat and murine lymphatic valves,^11,13^ we demonstrated that lymphatic vessel tone, which is modest (10-20% of the passive diameter), can reduce the trans-valvular pressure gradient required for valve closure. In veins that develop substantial tone (up to 80%), this effect is much more pronounced such that VV closure is prevented when a threshold value of venous tone is lost, ie, there no longer remains a physiological value of ΔP at which the valve will close. Thus, when spontaneous tone develops, only ∼14% of cervical VVs are incompetent compared to ∼60% incompetent VVs in the complete absence of tone. Even though our results apply only to mouse VVs, and to cervical VVs in particular, they establish a principle by which venous tone can determine if VVs are competent. Whether this concept extends to other VVs and to human VVs remains to be tested in subsequent studies. To our knowledge, no comparable studies of human VVs have been conducted and our own attempts to obtain viable samples of human veins containing VVs at our institutions have been unsuccessful. In other laboratories, various mechanical properties of human veins have been studied post-mortem, and occasionally in the absence of fixation,^7^ but based on our experience with human lymphatic vessels,^22^ any veins not immediately removed during surgery and maintained in cold physiological saline until cannulation and study would not be expected to develop spontaneous tone. It is possible that human VVs may not develop comparable levels of tone, or may have longer leaflets that provide more protection from loss of tone, or may be adapted to function properly under higher pressures/diameters, but these possibilities await further testing.

### A Detrimental Feed-Forward Mechanism

**Figure 6.**
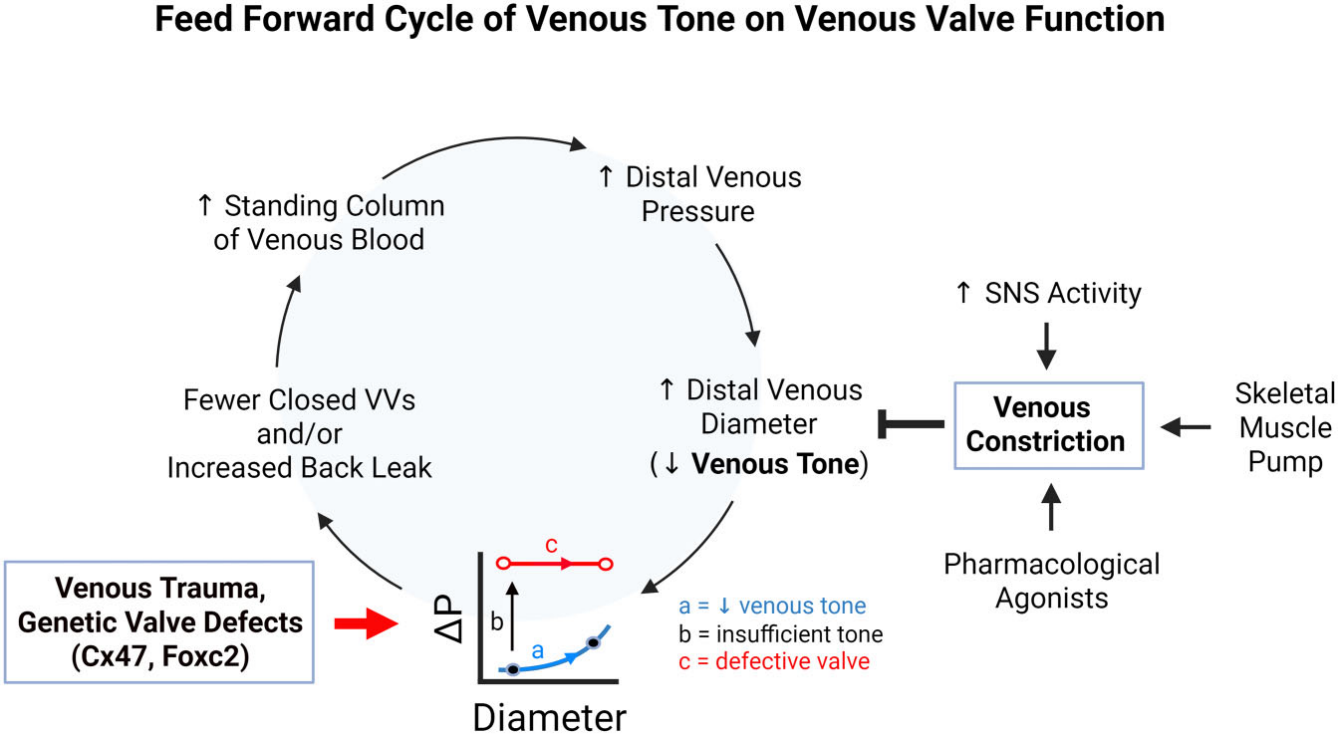
Feed forward cycle of venous tone on venous valve function during standing. Hypothesized mechanism by which loss of venous tone would lead to impaired VV closure and/or increased VV back leak, thereby increasing the standing column of venous blood and further impairing venous tone. These effects are normally counteracted by spontaneous (myogenic) tone, neural-mediated venous constriction and the action of the skeletal muscle pump (to compress veins and push blood through VVs). Venous tone and hence VV closure might also be enhanced by pharmacological agonists. The graph at the bottom of the cycle depicts relationships shown experimentally in Figure 5. Arrow “a” represents changes in venous tone associated with small changes in ΔP as a shift along the blue curve describing a “normal” valve. Arrow “b” represents the loss of sufficient venous tone to maintain normal valve gating that results in a shift to a different curve (blue to red), after which no physiological value of ΔP can close the valve. Arrow “c” represents valve behavior when trauma to the vein compromises tone or a genetic defect in the valve results in partial or complete valve incompetence. ΔP = the adverse trans-valve pressure gradient required for valve closure. SNS = sympathetic neural system. Created and licensed in BioRender. Davis, M. (2025) https://BioRender.com/u764qck

We hypothesize a general mechanism whereby loss of venous tone leads (1) to impaired VV closure and/or increased VV back leak, (2) thereby increasing the standing column of venous blood, which (3) further impairs venous tone, and (4) makes VV closure increasingly difficult or impossible. This progression is normally counteracted by spontaneous (myogenic) tone,^23^ neural-mediated venoconstriction and the action of the skeletal muscle pump to compress veins and push blood through VVs.^24^ The relationships between these variables are depicted in Figure 6. The graph at bottom of the cycle illustrates the relationship between the relative venous diameter and the ΔP required for VV closure shown experimentally in this study, where arrow “a” represents changes in venous tone associated with relatively small changes in the adverse ΔP required for closure as a shift along the blue curve describing “normal” valve function. Arrow “b” represents a shift to a completely different curve (blue to red) after loss of sufficient venous tone to maintain normal valve gating; along the latter (red) curve no physiological value of adverse ΔP is able to close the valve. Extrapolation of our findings suggests that venous tone and VV closure are likely to be enhanced by administration of appropriate pharmacological agonist(s) in this case. Arrow “c” in the graph represents conditions in which trauma to the vein (eg, a compromised wall) or valve (eg, clot, thrombectomy) or genetic defect in valve formation (caused by *Gja4*,^19^  *Gjc2*,^25^  *EPHB4*,^2^ or *Rasa1* ^16^ loss-of-function) results in complete valve incompetence. Increased venous tone may or may not be able to ameliorate valve incompetence in those cases, depending on the severity of the defect(s). For example, *Rasa1* loss-of-function results in shortening of VV leaflets,^16^ but if shortening is not too severe the leaflets might be able to overlap sufficiently to close the valve at low relative diameters, similar to the behavior of the partially incompetent valves in Figure 5A.

We were unable to test whether venous tone influences VV gating in other veins of the mouse. For unknown reasons, VVs are prominent in only a few selected veins of the mouse^19^ and it is possible that an extensive network of VVs is not required in quadrupeds such as the mouse. We would have preferred to study VVs from veins in the extremities, which presumably have comparable or greater tone (and sympathetic innervation) than veins in the upper thorax and neck. However, our preliminary tests revealed that other VVs were either not consistently present (eg, the proximal saphenous vein), incompetent (the VV in the brachial vein and VVs in tributaries of the proximal caudal femoral vein), or not amenable to study with our methods due to the number and/or tight spacing of side branches (the lower saphenous vein and its tributaries). Thus, whether other mouse VVs have similar properties to the cervical VVs described here is not yet known.

The mechanism by which tone facilitates VV gating is presumed to be similar to that for lymphatic valves.^11^ No evidence suggests that gating is anything but a passive process as no contractile smooth muscle fibers have been observed to insert into the leaflets of either lymphatic^26^ or VVs^16^ (at least in mice). Increased venous tone presumably relieves tension on VV leaflets such that a lower trans-valvular ΔP will induce closure, as predicted by numerical modeling studies.^27,28^ Although VVs have a much larger number of endothelial cells in their leaflets than lymphatic valves (∼150 versus ∼15)^13,16^ the ratio of leaflet length to vessel diameter is apparently the factor that dictates whether a valve has the potential to close.

### A Potentially New Therapeutic Approach

In humans, venous diseases are common and often involve venous wall and VV dysfunction.^6^ Venous dilation is characteristic of chronic venous insufficiency and can progress to the point where VV leaflets are pulled sufficiently apart as to prevent their overlap and permit back flow.^10^ VV leaflets can become deformed or perforated due to chronic venous distention or as a consequence of traumatic procedures such as thrombectomy.^29^ Leaflet closure can also be impaired by attached thrombi,^9^ thereby inducing various degrees of valve incompetence. Incompetent VVs will increase the pressure load on more distal valves and their associated venous walls by increasing the standing column of proximal blood.^24^ VVs do not regenerate or self-repair and the only remedies to date are valvuloplasty, valve transplantation or implanted devices to restore valve function.^30,31^

A number of physiological and pathological conditions result in increased sympathetic neural outflow to veins in the extremities.^24^ Our findings predict that venoconstriction, in addition to reducing venous capacitance, will act to protect and preserve VV competency, which is critical to interrupting the standing column of venous blood that develops in upright humans. Loss of neural tone or weakening of force development by venous smooth muscle will compromise VV function, but this can potentially be compensated by the administration of pharmacological agonists to enhance venous tone. Current use of pharmacotherapy in the context of chronic venous insufficiency is directed at pain relief or prevention of clotting and/or leukocyte-endothelium interactions.^32^ More extreme measures, such as thermal ablation or sclerotherapy,^33^ are of limited benefit as they divert venous blood to other routes, increase resistance of the overall venous network and increase the load on alternative routes of venous return,^6^ which may already be close to their threshold for being compromised. Our findings, if applicable to human VVs, suggest that pharmacological enhancement of venous tone may be a novel way to therapeutically target chronic venous insufficiency.

## Conflict of Interest

The authors have no conflicts of interest to report, financial, or otherwise.

## Data Availability

All data needed to evaluate the conclusions in the paper are preset in the paper. Data will be made available from the corresponding author upon request.
